# The Use of Cannabis and Its Effects on Postpartum Depression

**DOI:** 10.7759/cureus.27926

**Published:** 2022-08-12

**Authors:** Oghenetega E Ayisire, Okelue E Okobi, Ngozi J Adaralegbe, Adeyemi A Adeosun, Divyaanshi Sood, Nkemputaife P Onyechi, Ogochukwu Agazie, Hameed O Shittu, Zainab Akinsola, Chukwudike G Nnaji, Oluwasayo J Owolabi, Nneka J Umeh, Imolikhe C Imobighe, Adewale M Adedoyin, Madinah Usman

**Affiliations:** 1 Psychiatry, University of South Wales, Pontypridd, GBR; 2 Family Medicine, Lakeside Medical Center, Belle Glade, USA; 3 Allied Health Sciences, University of Connecticut, Waterbury, USA; 4 Molecular Pharmacology and Experiment Therapeutics, Mayo Clinic, Rochester, USA; 5 Oncology, Dayanand Medical College and Hospital, Ludhiana, IND; 6 Internal Medicine, University Hospitals Cleveland Medical Center, Cleveland, USA; 7 General Physician, College of Medicine University of Lagos, Idi Araba, NGA; 8 Internal Medicine, Federal Medical Centre, Abeokuta, NGA; 9 Internal Medicine/Family Medicine, Windsor University School of Medicine, Toronto, CAN; 10 Internal Medicine/Family Medicine, Windsor University School of Medicine, Chicago, USA; 11 Psychiatry, Lugansk Sate Medical University, Lugansk, UKR; 12 General Medicine, Brooklyn Queens Nursing Home, New York City, USA; 13 Obstetrics and Gynecology, Irrua Specialist Teaching Hospital, Irrua, NGA; 14 Internal Medicine, Lagos State University Teaching Hospital, Ikeja, NGA; 15 Medicine, Kharkiv National Medical University, Kharkiv, UKR

**Keywords:** depression, peripartum depression, tetrahydrocannabinol (thc), post partum depression, cannabis use

## Abstract

Cannabis use and depression management have been studied, with a preponderance of exacerbating effects, but there are few studies on postpartum depression (PPD). Depression affects a significant number of women, with a portion of it manifesting as PPD in childbearing women in the United States each year. The pharmacologic management approaches have disadvantages such as side effects, cost-benefit ratio, contraindications, use reluctance, medication adherence, and stigmatization in patients. Anecdotal claims of medical cannabis' therapeutic benefits have led to widespread legalization in several regions, making cannabis and its extracts a possible alternative. Cannabis is widely used during pregnancy and in general. Even though substance use disorders exacerbating depression symptoms have been reported, there are increasing reports and evidence about the therapeutic benefit of dose-dependent cannabis or its extracts in some depression symptoms, such as acute psychosocial stress relief, its purported anxiolytic effect, appetite, and sleep quality, thus stimulating more interest that may be inferred to depression. PPD marijuana use is unclear. This paper reviewed works of literature that claimed cannabis' therapeutic benefit in treating depression and, by extension, PPD. Our findings show the link between cannabis and PPD has not been fully explored. Self-reported studies link marijuana uses to positive mood, anxiety relief, sleep regulation, nausea and vomiting reduction, and appetite stimulation-all PPD symptoms. Others opposed postpartum marijuana use.

## Introduction and background

While the relationship between cannabis use and depression management has been studied and reported, with a prevalence of exacerbating effects on depression, there has been a dearth of studies documenting the effect of cannabis use on postpartum depression (PPD). PPD, now referred to as peripartum depression by the DSM V manual [1], is defined as the onset of depressive symptoms within four weeks of delivery, with other guidelines extending the onset period to six to twelve months postpartum [1-5]. In a more simplified term, depression with childbirth is termed PPD. These symptoms, which are frequently screened using the Edinburgh Postnatal Depression Scale, range from major to minor symptoms that meet the criteria for depression diagnosis, such as poor sleep, poor concentration, irritability, mood swings, guilt, feelings of extreme sadness, indifference, and anxiety, as well as changes in energy, poor appetite, and other subtle symptoms of depression that are generally ignored and dismissed as hormonal changes post-pregnancy. Depression is one of the most common psychiatric disorders, with an incidence twice as common in females and as common as one in eight women [6], while PPD affects between 15% and 20% of childbearing women each year, resulting in 600,000-800,000 cases of peripartum depression each year in the US [7]. The patho-etiology of PPD remains unknown but may include neuropsycho-endocrine factors like the hormonal imbalance after childbirth, exacerbation of external stressful factors like sleep deprivation that alters circadian rhythm, problems associated with lactation, the emotional stress of taking care of a newborn baby, and lack of support and help for managing work and childcare, malnutrition (Vitamin D deficiency), proinflammation, immune dysfunction, and the complex relationship with levels of leptin [1,8]. Treatments for PPD include pharmacotherapies like anti-depressants, psychotherapies, self-care education, and nutritional support [5,9,10]. These management approaches, with their cons like side effects, cost-benefit ratio, contraindications, use reluctance, medication adherence, and stigmatization in patients, have influenced more quest to search for other possible alternative management options. With the increasing anecdotal claims of medical cannabis's therapeutic benefit, which has led to the widespread legalization of cannabis in several regions, the topic of cannabis and its extracts as a possible alternative has gained traction [11]. Cannabis is a widely used psychoactive substance both during pregnancy and in the general population [12-14]. Even though the role of substance use disorders in exacerbating depression symptoms has been reported, there is an increasing number of reports [15-17] and evidence about the therapeutic benefit of dose-dependent cannabis or its extracts in some symptoms that make up the spectrum of depression, such as acute psychosocial stress relief, its purported anxiolytic effect, appetite, and sleep quality, thus stimulating more interest that may be inferable to depression and then women with PPD [17-19]. Marijuana use in PPD is still largely understudied. Given the scarcity of research on the use of marijuana as an alternative to approved management approaches, this paper aims to investigate the growing body of literature that claims cannabis's therapeutic benefit in treating depression and, by extension, PPD in non-breastfeeding mothers.

The objective of the study

Review existing literature and possible effects of marijuana on PPD documented in published experimental-controlled trials from 1985 to 2022.

The method of literature search

The literature search methodology has been used in this study to retrieve and review the literature relevant to the use of cannabis or marijuana for treating PPD from the literature of 1985 to 2022.

**Figure 1:**
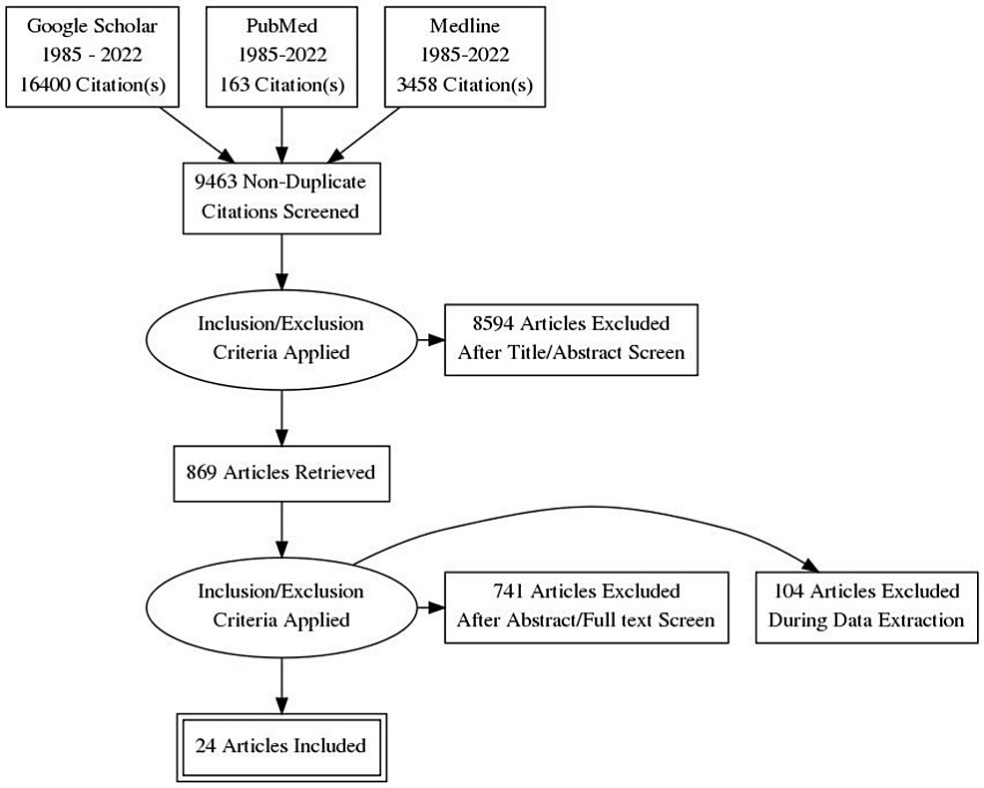
Prisma flow

The literature search strategy was based on the use of online databases such as Google Scholar, PubMed, and Medline by using the keywords such as “cannabis,” “marijuana,” “postpartum depression,” “peripartum depression,” and “depression" and "anxiety,” and “effects of cannabis.” These keywords and Boolean operators of AND, OR, and NOT were also used to narrow down the search results. The search results were then reviewed and analyzed. However, distinct inclusion and exclusion criteria were developed for the literature so that only relevant studies could be used for this literature review. The inclusion and exclusion are shown in Table 1. The prisma flow is shown in Figure 1, and the list of included studies is in the appendices.

**Table 1:** Inclusion and exclusion criteria

Inclusion criteria	Exclusion criteria
1) Literature relevant to the use of cannabis in managing postpartum depression	1) The studies that were relevant to the use of cannabis for medical conditions other than PPD were excluded because the objective of the study was focused on the use of cannabis for PPD.
2) The studies must be primary studies in which the effects of cannabis on treating PPD can be identified through experimental control.	2) Opinion pieces and non-scholarly articles, secondary studies, scoping reviews, meta-analyses and research approaches other than primary studies were excluded.
3) Human studies	3) Animal studies
4) The studies must be published in a peer-reviewed journal to maintain validity and reliability of the studies.	4) The studies that were published in non-peer-reviewed journals, and dissertations, were excluded.
5) The studies must be originally published in English for readability by the reviewers.	5) The studies that were originally published in a language other than English were discarded.
6) Works of literature published within the last ten years (1985–2022).	

## Review

An overview of the mechanisms of cannabis and theories of depression

Efforts to understand the mechanism of action of cannabis are still evolving. What is clear is that cannabis contains several psychoactive and non-psychoactive substances, some of which are the Δ-9-tetrahydrocannabinol (THC), cannabidiol (CBD), terpenes, flavonoids, etc. of THC and CBD, which exerts their effect on the brain by binding to a G-protein coupled receptor, gene-encoded by chromosome 6 known as the CB1 & CB2 cannabinol receptor [20,21]. To describe the mechanism of action of cannabis, signaling pathways were identified, explaining how this endocannabinoid system (ECS) regulates bodily functions [20-22]. This THC, CBD, and G-protein coupled signaling are present in both inhibitory GABAergic neurons and excitatory glutamatergic neurons of the central and peripheral nervous system [20-23]. The identification of this receptor suggests that endogenous cannabinoids, which function as physiological ligands, may exist in the brain [20-23]. These receptors interact with the psychoactive substances found in cannabis and exert physiological and psychological functions such as sleep, appetite, mood, and pain. On the other hand, there are several hypotheses about depression and its pathophysiology, ranging from deficiencies in monoamine neurotransmitters (norepinephrine [NE], 5-HT, and/or dopamine) to other factors like synaptic transport protein dysfunction, abnormalities in receptor structure, number, and functions. Other additional theories that seek to explain depression include several endocrine processes like dysfunctions in hormonal interplay, especially the hypothalamus-pituitary-adrenal axis (HPA), the hypothalamus-pituitary-ovarian axis (HPO), cortisol, growth hormone, thyroid hormones [24-26]. Attempts to explain the lack of concentration, anhedonia, interest, energy, and other symptoms of depression have been explored in various neurobiological imaging studies within the brain's limbic system [27]. Several of these biological and neurological models have been validated retrospectively by pharmacological treatment with antidepressant compounds. 

In postpartum, relatively few studies have examined the pathophysiological aspects of PPD compared to the extensive epidemiological literature on the condition. A couple of theories to explain the pathogenesis of PPD has been postulated. A few examples are the rapid drop in the endocrine concentration of pregnancy-related hormones, an alteration in circadian rhythm, levels of leptin and serotonin, the upregulation of proinflammatory cytokines, and stress induce HPA and HPO abnormalities [3,28-31]. However, the exact mechanisms of interplay between these theories of depression in the peripartum and the cannabinol signaling pathways are unclear because of the complexities in the regulation of CNS functions [3,25,26,29-33]. Several hypotheses, however, have been suggested. There has been some evolving evidence showing that CBD inhibits the uptake of these monoamine substances (serotonin, noradrenaline, dopamine, and GABA), which are thought to facilitate the anxiolytic properties of CBD. Perhaps the CB1 receptor is responsible for altering cognitive functions and mood by inhibiting the adenylate cyclase-based metabolite system. At the same time, the CB2 receptor controls dopamine release in the blood, which has psychoactive abilities and is responsible for reducing stress and anxiety [32-34].

An overview of cannabis for PPD

The postpartum period is stressful, especially for primigravidas, when compounded with socio-demographic risk factors, putting them at risk of anxiety and depression. While the prevalence of cannabis use in the peripartum period has increased [35-37], there has been a growing number of anecdotal reports contrasted with a paucity in the number of confirmations from clinical trials and meta-analyses on the evidence of the use of cannabis and depression. It is still unclear exactly how endogenous cannabis affects PPD physiologically. However, There have also been pockets of claims about the advantages of its extracts to managing some symptoms of PPD as individual entities - anxiolysis, loss of appetite, decreased energy, and poor sleep. The majority of these researches on the components of PPD and how cannabis affects PPD are either self-reported, retrospective, or observational. There was no documented clinical evidence on depression in humans as an “entity” contrarily, some reports suggested that cannabis use may contribute adversely to other components of major depression, peripartum depression, like increased reports of self-harm, or homicidal ideation towards the baby, agitation, associated depressive symptom, along with tobacco and alcohol [37-40]. With this wide spread of cannabis use in the general population, researchers are beginning to ask if there could be a positive effect, if any, on depression as an entity rather than the self-reports about its component. A few animal-based studies have documented the potential of using cannabis to treat anxiety and mood disorders [39]. The claims about cannabis's effect on PPD tend to have a symptomatic approach in management with varying degrees of success in several human populations rather than holistically. Most of these claims were also outside the postpartum subset.

Anxiety

Several complex dose-response interactions of the components of cannabis in the cortical glutamatergic and GABAergic terminals play a role in either anxiogenesis or anxiolysis, with low doses as anxiolytic and high doses inducing adverse effects [41]. However, there have been significant inconsistencies in the success of cannabis in suppressing anxiety disorders [42]. However, what is known is that stress impairs endocannabinoid signaling [41]. In some self-reported studies, cannabis was shown to be effective in controlling anxiety disorders [24]. For example, a self-reported observational cohort study surveyed 538 participants and compared baseline anxiety and depression differences between cannabis users and their cohort. They concluded that medicinal cannabis use might reduce anxiety and depressive symptoms in clinically anxious and depressed populations. However, the study did not say what proportion of its participants suffered from PPD. Similarly, cannabis's role as an anxiolytic has also been studied in several other studies. For example, in a study that assessed the effectiveness of cannabis in treating anxiety, study subjects rated cannabis as highly effective overall for treating anxiety with an average score of 8.03 on a Likert scale of 0 to 10 [43]. That study, with a total of 442 participants (173 were women), did not indicate the proportion of postpartum women in their findings.

Sleep

Poor sleep and various other forms of sleep disorders are also common features of PPD, and there have been attempts to use cannabis to manage insomnia. However, the mechanism of cannabis and sleep disorders is difficult to summarize for a variety of reasons. First, the majority of the studies were self-reported, combined with unknown cannabinoid concentrations that may have varying degrees of putative sedation. This potential role for THC has been described in treating several sleep disorders [44] like restless leg syndrome and narcolepsy, with the suggestion that THC may have applications in promoting the suppression of REM and increasing slow-wave sleep, and nabilone in managing PTSD-related nightmares. In a critical analysis published by Kuhathasan et al. [44], CBD in cannabis significantly improved REM sleep quality and prevented nightmares in women with PPD. However, none of these quantitative or qualitative documents have been weighed in a randomized controlled trial or during the postpartum period. In another study with a similar objective to analyze the effect of marijuana on sleep in 100 medically certified marijuana users, half of the respondents reported relief from insomnia and anxiety. In a different study with a comparable goal to examine how marijuana affects sleep, out of 100 medical marijuana users, half of the respondents reported relief from anxiety and insomnia.

Appetite stimulation

Other therapeutic benefits of cannabis have also been explored, albeit in peripartum. The roles played by the endocannabinoid systems and their documented effects on mood and appetite may be beneficial if weighed against blinded clinical trials. Loss of appetite, poor nutrition, and low vitamin levels may co-exist or characterize PPD. Another set of authors [45] analyzed national data from 2016-2017 using the Pregnancy Risk Assessment Monitoring System (PRAMS) and recruited 1147 women. They found that cannabis relieves nausea and vomiting, which are commonly prevalent among PPD women and pregnant women, which may, in turn, overcome some of the barriers to dietary intake during postpartum and improve nutrition. They found that cannabis relieves nausea and vomiting, commonly prevalent among women experiencing PPD and pregnant women. This, in turn, may overcome some of the barriers to dietary intake experienced by postpartum women, leading to improved nutrition. THC has a beneficial effect on the functioning of the gastrointestinal tract and decreases intestinal motility. Another retrospective cohort study by Richards et al. [46] reported that cannabis effectively increases appetite and regulates gastrointestinal functioning. A randomized-double blinded-placebo controlled study by Farokhnia et al. [47] explained that cannabis directly affects the regions of the brain that regulate hunger and stimulates the fasting system by stimulating appetite hormones such as ghrelin that signal the brain that it is time to eat. The study also mentioned that the THC contained in cannabis increases dopamine levels, enhances the pleasure of eating, and improves taste and smell, which affect a person's appetite. The study also mentioned that the THC found in cannabis raises dopamine levels, improves taste and smell, and makes eating more pleasurable, all of which affect a person's appetite.

Several other studies have attempted to use predictor models to determine the relationship between marijuana use and PPD. But these predictor models favored an association with depression rather than a therapeutic effect. For example, a study that looked at the frequency of PPD in marijuana users found that marijuana users with background depression had a 2.6-times increased risk of PPD. They did not find a statistically significant risk of PPD in marijuana users with no history of depression. Although they noted the effect confounders may have played in their finding [48], another retrospective cohort single-site study evaluated the association between continuous use of cannabis in pregnancy, generalized anxiety disorder, and PPD and found an increase in association [49]. Similar evidence supporting a positive correlation between cannabis use and PPD was documented in a longitudinal study done in Australia. The study done between 1989 and 1995 in 538 participants provided evidence that preconception cannabis use is associated with an increased risk of PPD [37].

Mother and child bonding

Mothers' detachment from their babies is frequently seen in PPD, which may progress to feelings of intense guilt, suicidal thoughts, and obsessive thoughts of harming the baby. Mother and child bonding is strengthened by breastfeeding. However, in people who use cannabis, the safety of cannabis in breastfeeding mothers remains questionable. Some studies [50-54] have reported that moderate amounts can be secreted in breast milk [52-55], causing significant side effects for the baby. This can adversely impact the growth and development of babies, restrict growth hormones, and slow down metabolic processes. These studies further showed that the active ingredients of cannabis are secreted into breast milk at about 0.8%-2.5%. They reported that cannabis causes small for gestational age, low birth weight, preterm birth, stillbirths, postpartum hypertension, preeclampsia, and eclampsia that can increase neonatal, maternal, and mortality. They also reported sedative effects, poor suckling, delayed motor development, and impact on brain function in breastfed babies of mothers who use cannabis, causing lower weight gain as compared to other children. Subsequently, traces of TCH remain in a child's bloodstream for up to six days if more than 9.47 ng/mL of cannabis is used during breastfeeding. Suicidal tendencies and self-harm are two of the significant symptoms present in PPD.

Suicide, self-harm, and homicidal ideation toward the baby****

Despite the fact that self-harm and suicidal ideation are symptoms of depression, there has been little research on it in the PPD period. The relationship between cannabis and self-harm has been documented in several studies with an overwhelming increased risk of self-harm. There is documented evidence that using cannabis during pregnancy worsens perinatal women's neuropsychiatric, behavioral, cannabis-related psychosis, and executive functioning issues. The exact mechanism is not clearly understood. This review did not find any randomized trial that studied or linked the use of cannabis and a reduction in self-harm in the PPD group. In a population-based retrospective cohort study done in Ohio that examined associations of substance use disorder with self-harm, suicide, and overall mortality risk in youths with mood disorders, the study enrolled 204,780 participants and concluded that it was significantly associated with self-harm [54]. Consistently, these studies did not disclose the proportion of their sample sizes that were women with PPD. 

Our view, limitations, and future

This review has summarized some views about the use of cannabis to manage PPD. Few or non-existing randomized trials have been done in humans to substantiate the numerous qualitative reports. The trials and evidence reviewed in this report are mostly self-reported, unblinded, and have small sample sizes. A critical evaluation of these studies also reveals a preponderance of biases from selection/sampling bias, design bias, measurement biases, response biases, and reporting biases, which invariably impacted the review outcome. Therefore, there is insufficient evidence to support the anecdotal claims about the effectiveness of using cannabis as a treatment option for PPD as an entity. However, the data are promising, and a need for a well-designed randomized clinical trial using standardized products of cannabis in this subset of the population with a unique presentation of depression. 

## Conclusions

Our findings show that the association between cannabis and its use in managing PPD has not been fully explored. In some self-reported studies, marijuana use was linked to positive effects on mood, anxiety relief, sleep regulation, nausea and vomiting reduction, and appetite stimulation-symptoms similar to PPD symptoms. Others strongly discouraged the use of marijuana during the postpartum period. However, we could not find any randomized clinical study involving humans that examined the use of cannabis for the treatment of PPD. Generally, cannabis is considered a recreational drug, which many clinicians discourage during pregnancy and postpartum. Therefore, as the call for expanding the legalization of marijuana increases, we call for caution. Further research is therefore needed to douse the anecdotal claims of the use of cannabis in managing PPD.

## Appendices

List of literature that met inclusion criteria:

1. Hyer et al. [8]

2. Emery et al. [12]

3. Martin et al. [24]

4. Bayrampour et al. [35]

5. Goodwin et al. [36]

6. Cao et al. [37]

7. Celestina et al. [38]

8. Melas et al. [39]

9. Young-Wolff et al. [40]

10. Rey et al. [41]

11. Ameringen et al. [42]

12. Kamal et al. [43]

13. Kuhathasan et al. [44]

14. Skelton [45]

15. Richards et al. [46]

16. Farokhnia et al. [47]

17. Mark et al. [48]

18. Ko et al. [49]

19. Brown et al. [50]

20. Shukla and Doshi [51]

21. Perez-Reyes and Wall [52]

22. Metz and Borgelt [53]

23. Marchand et al. [54]

24. Fontanella et al. [55]
